# The Role of Lifestyle and Psycho-Social Factors in Predicting Changes in Body Composition in Black South African Women

**DOI:** 10.1371/journal.pone.0132914

**Published:** 2015-07-14

**Authors:** Philippe Jean-Luc Gradidge, Shane A. Norris, Lisa K. Micklesfield, Nigel J. Crowther

**Affiliations:** 1 Centre for Exercise Science and Sports Medicine (CESSM), Faculty of Health Sciences, University of the Witwatersrand, Johannesburg, South Africa; 2 MRC/Wits Developmental Pathways for Health Research Unit, Faculty of Health Sciences, University of the Witwatersrand, Johannesburg, South Africa; 3 Department of Chemical Pathology, National Health Laboratory Service, Faculty of Health Sciences, University of the Witwatersrand, Johannesburg, South Africa; University of Alabama at Birmingham, UNITED STATES

## Abstract

**Background:**

This study aimed to determine whether lifestyle and psycho-social factors determine changes in body composition over 10 years in a population of black African females with a high prevalence of obesity.

**Materials and Methods:**

Data were collected from 430 women at baseline and 10-year follow-up. Dual energy x-ray absorptiometry-derived body fat mass and fat free soft tissue mass, and simple anthropometric measures were taken at both time points. Data on physical activity (PA), diet, smoking, and alcohol intake were collected at baseline. Body size dissatisfaction and body size discrepancy were determined at baseline using the feel minus ideal (FID) index and the perceived minus actual weight status discrepancy score (PAD), respectively.

**Results:**

All body composition measurements increased over 10 years (p<0.0005). Two distinct groups of overweight/obese females were identified using PAD and FID: one that was content with their body size and one that wished to be leaner. Vigorous PA at baseline was inversely associated with absolute changes in all measures of adiposity. In subjects who underestimated their body size at baseline (74.0% of the study population) changes in total and peripheral levels of body fat were less than in subjects who correctly identified their body size. In the group that underestimated body size, more women wanted to be leaner than in the group who knew their body size (60.1% vs 47.5%, p<0.05).

**Conclusions:**

Underestimation of body size is common and is associated with a lower gain in total body adiposity and a prevalent desire to lose weight.

## Introduction

Recent research has predicted that the proportion of overweight and obese women in developing countries such as South Africa will continue to rise, whereas the reverse applies to developed countries [1]. The increasing prevalence of overweight and obesity in the South African (SA) population is associated with an excessive burden of several non-communicable diseases such as type 2 diabetes and hypertension [2, 3]. Black SA women are more affected by obesity than men [4], a pattern that is common across the African continent and other developing countries [1]. Furthermore, data from the recent SA National Health and Nutrition Examination Survey shows that obesity is more prevalent in urban than rural black SA women (42.2% compared to 31.8%) [4], as urbanised women live in an environment that favours unhealthy dietary patterns [5], reduced physical activity (PA) [6, 7], and greater sedentary time [8]. However no studies have investigated whether these factors predict changes in body composition in an African population over time.

Psychosocial factors associated with obesity and body size preference specific to black SA women have been reported in cross sectional studies [9, 10]. These factors include social desirability to be fatter and a general tolerance of obesity [11]. Black SA women and adolescent girls have been shown to underestimate actual body size, indicating a high level of body size discrepancy, and have a low level of body size dissatisfaction [12, 13]. Culturally a larger body size is preferred as it is perceived to signify beauty, wealth, happiness, higher socioeconomic status, and ability to produce children [14], while thinness is associated with weakness, poverty and illness such as tuberculosis and human immunodeficiency virus [15]. Furthermore, a recent study has shown that the body image silhouette chosen by urbanised black female adolescents for their ideal body silhouette represents a higher body mass index (BMI) than that chosen by white female adolescents. Within the same study, when asked what body silhouette they perceive their families would prefer them to have, black participants chose a silhouette with a BMI that was higher than that chosen by the white participants [16]. This study also showed that a higher percentage of black girls had an EAT-26 (Eating Attitudes Test) score >20 (indicating the possibility of developing eating disorders such as anorexia nervosa, bulimia nervosa, or preoccupation with food), and that a higher proportion of black adolescents girls, compared to white girls, had greater body size dissatisfaction, and were more likely to control what they ate. Another SA study similarly suggested that acculturation may be occurring in young black females, and that there could be a conflict between traditional beliefs and Western ideals around body size [17]. Therefore, the aim of this study was three-fold: (i) to describe the change in body composition over a 10 year period in a cohort of urban black SA women; (ii) to determine whether baseline measurements of body size dissatisfaction and body size discrepancy are associated with baseline body composition measures, and correlate with changes in body composition variables over 10-years of follow-up, and (iii) to determine whether baseline lifestyle factors including diet and physical activity are associated with changes in body composition.

## Materials and Methods

### Study population

This longitudinal study included black SA urban-dwelling women from Soweto, Johannesburg. These women were caregivers of participants from the Birth to Twenty (Bt20) cohort, a study which started in 1990 with a sample of 3,273 subjects to examine the health and development of children [18, 19]. The majority (78.8%) of the sample were biological mothers of the Bt20 cohort, with the remainder being related to the child in some way. Baseline data were collected on all caregivers who were black African and > 18 years of age in 2002/3 (n = 1,251). Follow up data collection was completed 10 years later (2012/13) on 702 women, 428 of whom had anthropometric (weight and height) data at both time points. The Human Research Ethics Committee at the University of the Witwatersrand granted ethical clearance to perform this study and the participants provided written informed consent (M110627).

### Body composition

An electronic weighing scale (Dismedinc., Anjou, Canada) was used to measure total body weight (nearest 0.1 kg) and a wall mounted stadiometer (Holtain Ltd., Crosswell, UK) to measure standing height (nearest 0.1 cm) of the participants while dressed in minimal clothing and barefoot. Body mass index (BMI; kg.m^-2^) was calculated. Waist and hip circumferences were measured using an inelastic, flexible tape measure. Waist circumference (WC) was measured at the narrowest region of the torso whilst the participant stood erect with feet close together. Hip circumference (HC) was measured around the widest part of the buttocks just below the gluteal fold [20]. The measurements were performed by trained research assistants, all of whom had a coefficient of variation (CV) <1% for body weight and standing height measurements, and <2% for waist and hip circumference measurements.

Dual-energy X-ray (DXA) absorptiometry (Hologic QDR 4500A, software version 11.2, Hologic Inc., Bedford, Massachusetts, USA) was used to measure total body fat mass, central (trunk) and peripheral (arms and legs) adiposity, and fat free soft tissue mass (FFSTM) (CVs for DXA parameters were <2% for total fat mass, and 1% for fat-free soft tissue mass).

### Body size dissatisfaction and body size discrepancy

At baseline, drawings of nine female body silhouettes (adapted from Stunkard et al. [21]) ranging from ‘very thin’ (numbered as 1) to ‘very heavy’ (numbered as 9) were shown to participants and used to evaluate body size dissatisfaction and body size discrepancy. Body size discrepancy was assessed by calculating a discrepancy score between perceived and actual weight status (PAD) using a method adapted from Mchiza et al. and Zaccagni et al. [9, 22]. The participant chose a silhouette that they considered best represented their current weight. These were coded as follows: 1 = silhouettes 1 and 2 [underweight]; 2 = silhouettes 3–5 [normal weight]; 3 = silhouettes 6 and 7 [overweight]; 4 = silhouettes 8 and 9 [obese]). The PAD score was then calculated by subtracting the actual BMI status (coded using measured BMI as: 1 = underweight [<18.5 kg.m^-2^], 2 = normal weight [18.5–24.9 kg.m^-2^], 3 = overweight [25.0–29.9 kg.m^-2^], 4 = obese [≥30.0 kg.m^-2^]) from the code for the perceived weight status silhouettes. Negative PAD scores represent underestimation of weight status, positive scores represent overestimation of weight status, and zero scores represent participants who had an accurate perception of their weight status.

Body size dissatisfaction was measured by asking the participant to choose the silhouette that best portrayed how they wanted to look (known as the ‘ideal’ body shape). This was then subtracted from the ‘feel’ score (the silhouette that portrays what they perceive themselves to look like) to calculate the feel minus ideal (FID) score. Positive scores represent a desire to be leaner, a zero score represents contentment with body size, and negative scores indicate a desire to be fatter [10, 23]. The body size dissatisfaction and body size discrepancy scores were used to categorise the participants into negative, zero and positive groups of PAD and FID.

### Dietary behaviour, alcohol consumption and tobacco use

High fat consumption was measured at baseline using an adapted food frequency questionnaire from a previous SA study investigating factors associated with overweight and obesity [24]. The questionnaire determined consumption frequency of high fat foods in the past week including fatty cuts of red meat, chicken with skin, full cream milk, processed meats, crisps, and fried food items, e.g. fried eggs, fish and French fries, consumed on a regular basis (≥1 day per week). Alcohol intake was categorised as low (<1 drink/day), moderate (1–3 drinks/day), or high (>3 drinks/day). Current smoking status was also determined by questionnaire. All participants were asked if they had attempted to lose weight in the last 24 months by reducing their food intake, taking diet shakes/drinks, and/or joining organisations which focus on structured weight reduction programmes.

### Physical activity questionnaire

The Global Physical Activity Questionnaire (GPAQ), developed for global physical activity surveillance, was completed via interview to obtain self-reported physical activity at baseline [25]. Total moderate-vigorous physical activity (MVPA) in minutes per week (mins/wk), was calculated from the sum of occupation, travel-related (walking) and leisure time MVPA. Examples of moderate (e.g. mopping the floor at home) and vigorous (e.g. carrying a load on the head while walking uphill) intensity activities in these various domains were explained to each of the participants. Walking for travel was analysed individually as it was the most common form of physical activity observed in this study population. Sitting time (measured in mins/wk) was used as a proxy for sedentary behaviour.

### Socio-economic status

Asset ownership was used as a proxy measure for household socio-economic status (SES) at baseline [26]. The questionnaire was based on the ownership of twelve household commodities: electricity, television, radio, motor vehicle, refrigerator, washing machine, telephone, video machine, microwave, analogue television channel decoder for subscription television, satellite television, and mobile phone. The twelve household commodities were ranked in order of value and an overall SES score was then calculated using the ranks. The overall SES score ranged from 0 to 78. Level of education was also used as a measure of SES, and was categorised as no education (coded as ‘0’), completed primary school but did not attend high school (coded as ‘1’), attended high school but did not graduate (coded as ‘2’) and completed high school (coded as ‘3’).

### Statistical methods

Analyses were performed on the women for whom we had body composition and weight and height data at baseline and 10-year follow-up (N = 428). The Statistica software package was used for all statistical analyses (version 12, StatSoft, Tulsa, USA) [27]. Data that were not normally distributed (total MVPA, total walking for travel, vigorous physical activity, moderate physical activity, and total sitting time per week) were log transformed to normality. Continuous, normally distributed variables are presented as mean ± SD whilst data that was not normally distributed is presented as median (interquartile range [IQR]). Differences between baseline and 10 year data were assessed by Student’s paired t-test or Chi-square test. Variables were compared between the 3 FID, and between the 3 PAD groups, using ANOVA and followed by the Tukey *post hoc* test only in cases when the ANOVA was significant (p<0.05). A cluster variable was created using physical activity and consumption of fatty foods with ANOVA used to compare body composition across the following groups: physically inactive and consume high fat foods (n = 100), physically inactive and consume low fat foods (n = 19), physically active and consume high fat foods (n = 240), physically active and consume low fat foods (n = 33).

Multiple linear regression analyses were conducted to determine if baseline (predictor) variables were associated with absolute changes in the body composition (outcome) variables (FFSTM, waist circumference, BMI, central adiposity, peripheral adiposity, and total body fat mass) 10 years later after adjusting for potential confounding variables, i.e. age, SES score, education, and the respective baseline body composition variables. Results of the multiple linear regression models are presented as standardised β values to enable direct comparison of the strengths of the associations. Prior to performing multiple regression analysis, simple bivariate analyses were performed to determine which of the following baseline (predictor) measures [total vigorous physical activity, total walking for travel, alcohol use, cluster variable for physical activity status and consumption of fatty foods, smoking status, weight loss practices, FID score and PAD score] were associated with the various dependent variables, and those with p<0.05 were included in the multivariate regression models along with the four possible confounding variables described above. Only the independent variables that had significant (p<0.05) β values are reported in the results section. Collinearity between independent variables was assessed using the Variance Inflation Factor (VIF), and no collinearity was observed (all VIFs<2.0). Dummy variables were generated for the following variables: FID and PAD scores, the cluster variable for physical activity and consumption of fatty foods, education, smoking status, and alcohol consumption. The reference groups for each of these variables were as follows: zero FID, zero PAD, physically active and low fat consumption, no education, non-smokers, and people who do not consume alcohol. Significance was accepted at an alpha level of p≤0.05.

## Results

### Characteristics of the study cohort

Mean age at baseline was 41.1 ± 5.35 years and at follow-up age was 49.3± 5.33 years. Within the study cohort 49.5% of participants had completed high school. Complete DXA data was not available at both time points for all subjects due to the fact that not all study subjects were able to perform DXA scans at the baseline visit on weekdays due to work commitments and we could only accommodate a small number of subjects for data collection on weekends. Women for whom DXA data was available at both time points (n = 264) were slightly older, but showed no differences for any of the other measured variables when compared to women without (n = 164) DXA data (S1 Table). There was a significant increase in all body composition measures between baseline and follow up (all p<0.001), with a mean weight gain for the whole sample of 5.17 ± 8.86 kg (Table 1). The prevalence of a high waist circumference (≥80cm) and obesity (BMI≥30kg.m^-2^) increased significantly between baseline and follow-up (both p<0.001).

**Table 1:** Body composition characteristics at baseline and at 10-year follow-up.

Variable	N^a^	Baseline	Follow-up	Percentage change
**Weight (kg)**	428	77.4 ± 17.3	82.6 ± 18.9*	7.13 ± 12.1
**Body mass index (kg.m** ^**-2**^ **)**	428	30.8 ± 6.70	33.1 ± 7.36*	7.98 ± 11.9
**Waist circumference (cm)**	415	87.7 ± 13.2	98.5 ± 14.7*	12.8 ± 11.4
**Hip circumference (cm)**	413	114 ± 13.7	119 ± 15.2*	4.09 ± 7.44
**Waist-to-hip ratio**	413	0.77 ± 0.08	0.83 ± 0.08*	8.65 ± 9.96
**Fat mass (kg)**	264	29.8 ± 10.2	32.9 ± 10.6*	13.6 ± 23.75
**Fat free soft tissue mass (kg)**	264	38.5 ± 5.86	45.0 ± 7.29*	17.2 ± 7.99
**Central adiposity (kg)**	261	13.4 ± 5.28	14.4 ± 5.33*	11.6 ± 28.7
**Peripheral adiposity (kg)**	261	16.4 ± 5.49	17.7 ± 5.84*	9.8 ± 20.5
**Obesity (BMI≥30 kg.m** ^**-2**^ **)**	428	50.9 (46.2, 55.7)	65.8 (61.4, 70.4)*	29.3^†^
**Waist (≥80cm)**	415	70.6 (66.2, 75.0)	89.9 (87.0, 92.8)*	27.3^†^

Data presented as mean ± SD for continuous data and % (95% CIs) for categorical data; ^**a**^N at baseline and at 10-year follow-up

^*^p<0.001 versus baseline values

^†^Formula for percent change in prevalence: (follow-up prevalence–baseline prevalence)/baseline prevalence.

### Body size perception and body size discrepancy

At baseline, 10.5% of subjects wanted to be fatter (negative FID score), 57.4% wanted to be leaner (positive FID score), and 32.1% were content with their body image (zero FID score; Table 2). The proportion of women in each of these groups did not change significantly at follow-up, i.e. 9.1% subjects wanted to be fatter, 53.7% wanted to be leaner, and 37.2% were content with their body size. At baseline, women who wanted to be leaner had a significantly higher BMI, waist and hip circumferences, central and peripheral adiposity, and total fat and fat free soft tissue mass, than those with a negative or zero FID score (p<0.0005 for all comparisons). Similar differences were observed at follow up (S2 and S3 Tables). There were no differences between the FID groups for absolute change in the any of the body composition measures even when this data was expressed as percentage change from the baseline, and when FID negative and zero groups were combined and compared with the FID positive group.

**Table 2:** Comparison across baseline FID groups of baseline body composition measures and absolute change in body composition variables over 10-year follow-up.

Variable	Subjects who wanted to be fatter	Subjects who were content with their body size	Subjects who wanted to be leaner
**Proportion in each FID group (%)** ^**b**^	10.5% (45)	32.1% (138)	57.4% (247)
**Age (years)**	42.2 ± 5.84 (45)	42.1 ± 5.49 (138)	40.3 ± 5.05 (247)
**BMI (kg.m** ^**-2**^ **)** ^**b**^	25.1 ± 4.16 (44)	28.4 ± 4.91 (137)^†^	33.2 ± 6.85 (247)^†††^ ^***^
**Absolute change in BMI (kg.m** ^**-2**^ **)**	1.46 ± 2.99 (44)	2.60 ± 3.54 (137)	2.31 ± 3.54 (247)
**Waist circumference (cm)** ^**b**^	77.2 ± 8.21 (45)	83.4 ± 10.4 (136)	91.8 ± 13.6 (243) ^†††^ ^***^
**Absolute change in waist circumference (cm)**	10.5 ± 8.11 (43)	11.1 ± 8.91 (134)	10.7 ± 10.0 (238)
**Hip circumference (cm)** ^**b**^	102 ± 9.28 (45)	110 ± 10.8 (136)^†^	119 ± 13.8 (242) ^†††^ ^***^
**Absolute change in hip circumference (cm)**	2.98 ± 6.08 (43)	4.58 ± 7.42 (133)	4.69 ± 9.86 (237)
**Fat mass (kg)** ^**b**^	20.2 ± 6.35 (33)	28.0 ± 9.29 (87)^†^	33.4 ± 10.4 (157) ^†††^ ^*^
**Absolute change in fat mass (kg)**	4.02 ± 6.04 (32)	3.53 ± 6.10 (84)	2.72 ± 5.89 (148)
**Fat free soft mass (kg)** ^**b**^	35.9 ± 5.10 (33)	37.1 ± 5.18 (87)	40.3 ± 6.38 (157) ^†††^ ^***^
**Absolute change in fat free soft mass (kg)**	5.78 ± 2.99 (32)	6.49 ± 2.88 (84)	6.80 ± 3.26 (148)
**Central adiposity (kg)** ^**b**^	8.68 ± 3.34 (32)	12.3 ± 4.68 (86)	15.2 ± 5.29 (155) ^†††^ ^*^
**Absolute change in central adiposity (kg)**	1.56 ± 2.88 (30)	1.15 ± 3.20 (84)	0.79 ± 3.10 (147)
**Peripheral adiposity (kg)** ^**b**^	11.5 ± 3.56 (32)	15.6 ± 5.05 (86)	18.1 ± 5.82 (156) ^†††^ ^*^
**Absolute change in peripheral adiposity (kg)**	1.64 ± 2.67 (30)	1.48 ± 3.06 (84)	1.29 ± 3.46 (148)

Data presented as mean ± SD (n)

^†^P<0.05

^†††^P<0.0005 versus subjects who wanted to be fatter

^*^P<0.05

^***^P<0.0005 versus subjects who were content with body shape

^**b**^ baseline values.

With regard to body size discrepancy at baseline, 74% of the subjects underestimated their actual body size (negative PAD score), 2% overestimated actual body weight (positive PAD score), and 24% correctly perceived their actual body weight (zero PAD score; Table 3). At follow up, the proportion of women in each of the PAD groups were similar to baseline, i.e. 73.5%, 0.70%, and 25.8% respectively. Women who underestimated actual body weight status had a significantly higher BMI, waist and hip circumferences, and total fat and fat free soft tissue mass, than those who overestimated or correctly perceived their actual body weight (p<0.0005 for all comparisons). Women who correctly perceived actual body weight had a significantly greater increase in fat mass than the women who underestimated body weight.

**Table 3:** Comparison across baseline PAD groups of baseline body composition measures and absolute change in body composition variables over 10-year follow-up.

Variable	Subjects who underestimated actual body size	Subjects who accurately perceived actual body size	Subjects who overestimated actual body size
**Proportion in each PAD group (%)** ^**b**^	74% (316)	24% (103)	2% (9)
**Age (years)**	41.3 ± 5.41 (316)	40.3 ± 5.18 (103)	42.4 ± 4.64 (9)
**BMI (kg.m** ^**-2**^ **)** ^**b**^	32.6 ± 5.74 (316)	26.2 ± 6.89 (103)	21.5 ± 2.39 (9) ^†††^ ^***^
**Absolute change in BMI (kg.m** ^**-2**^ **)**	2.17 ± 3.60 (316)	2.80 ± 3.66 (103)	1.81 ± 2.47 (9)
**Waist circumference (cm)** ^**b**^	91.0 ± 11.9 (312)	78.6 ± 12.3 (101)	72.0 ± 6.93 (9) ^†††^ ^*^
**Absolute change in waist circumference (cm)**	10.4 ± 9.73 (307)	12.3 ± 8.79 (97)	10.6 ± 6.97 (9)
**Hip circumference (cm)** ^**b**^	117 ± 12.2 (312)	106 ± 13.8 (100)	94.4 ± 7.81 (9) ^†††^ ^***^
**Absolute change in hip circumference (cm)**	4.17 ± 9.14 (306)	5.54 ± 7.88 (96)	4.73 ± 5.98 (9)
**Fat mass (kg)** ^**b**^	33.3 ± 9.38 (209)	21.2 ± 7.98 (60)	15.2 ± 5.03 (7) ^†††^ ^***^
**Absolute change in fat mass (kg)**	2.57 ± 6.92 (198)	5.04 ± 5.99 (59) ^†††^	3.24 ± 3.88 (6)
**Fat free soft mass (kg)** ^**b**^	40.3 ± 5.66 (209)	34.8 ± 4.99 (60)	28.4 ± 2.59 (7) ^†††^ ^***^
**Absolute change in fat free soft mass (kg)**	6.89 ± 3.13 (198)	5.88 ± 2.87 (59)	3.86 ± 2.44 (6)
**Central adiposity (kg)** ^**b**^	15.6 ± 4.67 (206)	8.79 ± 4.02 (59)	6.33 ± 2.35 (7) ^†††^ ^***^
**Absolute change in central adiposity (kg)**	0.73 ± 3.10 (196)	1.85 ± 3.11 (58)	1.20 ± 2.28 (6) ^†††^
**Peripheral adiposity (kg)** ^**b**^	18.1 ± 5.26 (206)	12.3 ± 4.60 (60)	8.86 ± 3.21 (7)
**Absolute change in peripheral adiposity (kg)**	0.99 ± 2.98 (196)^*^	2.74 ± 3.93 (59)	1.25 ± 1.71 (6)

Data presented as mean ± SD (n)

^†^P<0.05

^†††^P<0.0005 versus subjects who underestimated actual body size

^*^P<0.05

^***^P<0.0005 versus subjects who accurately perceived actual body size

^**b**^baseline values.

There were 316 participants who underestimated their body size. These subjects had a high BMI (32.6 ± 5.74; see Table 3) and 60.1% of them wished to be leaner i.e. had a positive FID score, 8.20% wanted to be fatter, and 31.7% were content with their body size. The women who underestimated but were content with their body size were older (42.7 ± 5.28 vs 40.4 ± 5.16 years; p<0.05) and less obese (30.3 ± 4.01 vs 34.5 ± 5.78; p<0.0005) than those women who wanted to be thinner, and less of them completed high school compared to the latter group (46.0% vs 56.3%; p = 0.06). There were 103 participants who accurately perceived their body size, of whom 17.5% wanted to be fatter, 35% were content with their body size and 47.5% wanted to be leaner (for the latter value (47.5%), p<0.05 vs those subjects who underestimated body size and wanted to be lean (60.1%)).

### Lifestyle factors

The median (IQR) sitting time for all participants at baseline was 1260 (840–1890) minutes per week, and was significantly different (p<0.05) between the subjects who wanted to be fatter (1680 (840–2940) minutes per week) compared to the subjects who were content with their body size (1260 (840–1680) minutes per week) and the subjects who expressed a desire to be thinner (1260 (840–1680) minutes per week). For all participants the median time spent in walking for travel was 150 (60–300) minutes per week, for work MVPA was 0 (0–0) minutes per week, and for leisure MVPA was 0 (0–120) minutes per week. Total median MVPA was 350 (150–1240) minutes per week, total vigorous physical activity and total moderate physical activity were 0 (0–0) and 315 (150–1240) minutes per week, respectively. None of these physical activity variables measured at baseline were associated with body size discrepancy or body size dissatisfaction, and were also not significantly different between the PAD or FID groups.

The majority of participants (88.2%) reported consuming butter or margarine on bread, 55.9% reported eating chicken with skin, 48.6% ate processed meat, 33.7% ate red fatty meats, and 49.8% drank full cream milk on a regular basis (>1 time per week). A significantly higher amount of women who wanted to be leaner consumed fatty red meat compared to those women who wanted to be fatter (44.4% vs. 13.5%, p<0.05). The majority (97%) of the participants did not smoke, and 16.7% consumed alcohol on a regular basis (≥1 time per day), whilst 25.7% had participated in some form of weight loss strategy in the past 24 months. A significantly higher number of women who wanted to be leaner participated in some form of weight programme compared to those women who indicated a desire to be fatter (84% vs. 13%, p<0.0001). No significant differences were noted between the groups defined using the cluster variable (physical activity and fatty food intake) for changes in body composition (as determined by ANOVA). This cluster variable also did not contribute significantly to the multiple regression models shown in Table 4.

**Table 4:** Multiple linear regression analyses displaying the major predictors of change in body composition in black African women from Soweto.

Model number	Dependent variables	N	Independent variables	Beta coefficients (P-value)
**1**	Absolute change in waist circumference	382	Alcohol intake at baseline (>3 drinks/day):	-0.15 (0.003)
			Total vigorous PA (baseline):	-0.15 (0.002)
			Waist circumference (baseline):	-0.17 (0.001)
**2**	Absolute change in body mass index	430	Total vigorous PA (baseline):	-0.11 (0.02)
**3**	Absolute change in fat free soft tissue mass	241	Active smoker at baseline:	-0.14 (0.02)
			Age (baseline):	-0.14 (0.03)
			Fat free soft tissue mass (baseline):	0.21 (0.003)
**4**	Absolute change in total body fat	264	Subjects who underestimated actual body size (baseline):	-0.16 (0.01)
			Total body fat (baseline):	-0.22 (0.002)
			Total vigorous PA (baseline):	-0.12 (0.04)
**5**	Absolute change in central adiposity	260	Central adiposity (baseline):	-0.24 (0.001)
			Total vigorous PA (baseline):	-0.15 (0.01)
**6**	Absolute change in peripheral adiposity	261	Subjects who underestimated actual body size (baseline):	-0.15 (0.03)
			Total vigorous PA (baseline):	-0.13 (0.04)

Data presented as standardised beta coefficient (p-value).

### Multivariate regression

The multivariate regression models showing the contribution of various baseline factors to change in body composition are presented in Table 4, with age, SES, education and the respective baseline body composition variables included in each of the 6 models. Total vigorous physical activity and high alcohol consumption (≥3 drinks/day) were each inversely associated with absolute change in waist circumference (model 1). In the model for absolute change in BMI (model 2), total vigorous physical activity was the only significant independent variable. Smoking and age were inversely associated with change in FFSTM, while baseline FFSTM was positively associated (model 3). Although the prevalence of smoking was low (3%) an effect was seen on change in FFSTM and this is due to a strong effect of smoking as shown by the difference in change in FFSTM between smokers (n = 13) and non-smokers (n = 382) (3.03 ± 2.81 vs 6.69 ± 3.13, p = 0.003). Model 4 showed that total vigorous physical activity and baseline total body fat were both inversely associated with an absolute change in total body fat, which was lower in those who underestimated body size when compared to those who accurately assessed body size. Total vigorous physical activity and baseline central adiposity were both inversely associated with change in central adiposity (model 5). Total vigorous physical activity and an underestimation of body size were both negatively associated with absolute change in peripheral adiposity (model 6).

## Discussion

In this cohort of ageing black South African women we observed significant weight gain over a 10 year period, with an obesity prevalence of 65.8% at follow up, and with nearly 90% of the participants having a waist circumference ≥80cm. The most significant contributors to less change in the various body composition measures were the underestimation of body size at baseline, time spent doing vigorous physical activity, and alcohol consumption.

This study is the first to determine the contribution of body image to long-term body composition change in black SA women. Our study demonstrates that the majority (57.4%) of the women wished to be leaner, and that these women had higher baseline BMI, total body fat and fat-free mass, central and peripheral adiposity, waist and hip circumference values, than the women who wanted to be fatter and those who were happy with their body size. In contrast, previous cross-sectional studies have shown that African women have a general acceptance of overweight and obesity [11], with a larger body size traditionally being viewed as a symbol of beauty, wealth, happiness, and optimal fertility in African cultures [11, 14, 28], whereas leanness was associated with sickness and lower socioeconomic status [15]. Our data therefore suggests that within our cohort of adults the acceptance of female obesity within African traditions exists side-by-side with a more western ideal of a lower body weight. Within adolescent black females the situation is similar, with the study of Gitau et al. [16] showing that although black adolescent females are more comfortable with a body size that is higher than that selected by white adolescents females, the black females were more likely to have a predisposition toward eating disorders than their white counterparts. This again suggests a clash between the traditional African view of overweight/obesity as being the ideal female body state and the modern Western tradition of leanness as the ideal female body state [17].

This is the first study to have measured whether the discrepancy between actual and perceived weight status in SA women predicts future weight gain. The majority (74%) of women at baseline underestimated their weight and had significantly higher measures of all body composition parameters at baseline, when compared to the other groups. However these women had less absolute change in central and peripheral adiposity at follow up when compared to those subjects who had accurately estimated their body size. This may be related to the fact that a higher proportion of this group wanted to be leaner when compared to the subjects who accurately identified their body size. This may be advantageous for future obesity intervention programs. Other SA studies have also shown that black African females tend to underestimate their actual body size [12, 13]. However, 31.7% of the women who underestimated their body size were content with their weight status, even though their mean BMI was high at 30.3 ± 4.01. Fewer of these women had completed high school and were older than those women who wished to be leaner. These data suggest that within our study population there are two distinct groups of overweight/obese women who differ in terms of age, education and the desire to lose weight. These findings may be important when introducing obesity intervention programs into this population, with different approaches required for each group of women.

This is the first longitudinal study on the effect of physical activity on weight change in black African females. Our results showed that baseline total vigorous physical activity was inversely associated with change in BMI, waist circumference, total body fat, and central and peripheral adiposity. These results are comparable to those from a study conducted in middle-aged obese and sedentary American women which showed that higher intensity physical activity was more effective in reducing both intra-abdominal and subcutaneous fat than lower intensity physical activity [29]. Further, it has been observed that exchanging 1 hour of sitting time with light physical activity can significantly decrease both visceral and subcutaneous adipose tissue [30]. In our study, sitting time was significantly lower in those women who wanted to be leaner compared to those women who wanted to be fatter, but body size dissatisfaction and body size discrepancy were not associated with differences in the level of physical activity. This data suggests that body size dissatisfaction or discrepancy may not have an impact on physical activity behaviour in this population, which is contrary to one previous study which has shown that physical activity is perceived to be associated with thinness [13], and that body size dissatisfaction may modulate sedentary behaviour. Moreover, participating in vigorous physical activity provides additional advantages to health such as improved skeletal health and reduced risk of cardiovascular diseases [31]. However, only 10% of the women in our study participated in vigorous intensity physical activity, which is similar to the data for women from other developing countries [6, 32]. These data suggest that efforts to combat obesity in this population should include high intensity physical activity programmes.

Findings in other studies of alcohol consumption and body weight have shown that more frequent drinking is associated with leanness [33, 34], while binge drinking is associated with central obesity [35]. In comparison, our findings show that higher alcohol consumption was inversely associated with change in waist circumference. This finding is consistent with a large Danish longitudinal study which showed that the level of alcohol intake was inversely associated with change in waist circumference in women [36]. The authors attribute this result to a thermogenic effect, i.e. the action of alcohol dehydrogenase and the microsomal ethanol-oxidising system increasing thermogenesis in frequent alcohol drinkers. Our analysis also shows that smoking has an inverse association with change in FFSTM, implying that women in our cohort who smoke gain less muscle mass over time. Our findings also show that age was negatively associated with change in FFSTM, reinforcing the well documented loss of muscle mass with ageing [37]. The main result of premature sarcopenia would be a loss of functional capacity, particularly for activities of daily life that require muscle strength. Nicotine acts as an appetite suppressant and increases energy expenditure, which may explain the reason for smokers tending to have lower body weight than non-smokers, and why smoking cessation is followed by an increase in fat mass [38]. As active smoking was only observed in 3% of the cohort in our study, and because this is a cross sectional analysis causality should be inferred with caution. With regards to dietary behavior, we showed that many of the women in our study ate foods high in fat. In spite of this, the consumption of these foods did not influence long term weight gain. This is consistent with a large American study, which showed a weak non-significant correlation between weight change and whole fat dairy foods, but a strong negative correlation with nuts, vegetables and fruits [39].

In South Africa [12, 40, 41] and other sub-Saharan African countries [42, 43], SES has been shown to be positively associated with obesity in cross sectional studies, as has level of education [12, 40–43]. However, the findings from our study show that baseline measures of education or SES did not affect change in body composition. The reason for this finding is not known however one possible explanation may be the low variation in education and SES levels within this cohort. Socioeconomic status and alcohol intake were only measured at baseline in our study and therefore the effect of changes in these variables could not be assessed. However it is unlikely that SES would change sufficiently over the study period to modulate any of the outcome variables. It is possible that alterations in alcohol consumption over the 10 year period may have effects on body composition changes, but it must be noted that the prevalence of alcohol usage in this cohort was low.

This longitudinal study has a number of limitations including the use of self-reported physical activity and sedentary behaviour, derived from the GPAQ instrument. This questionnaire has the potential to overestimate both physical activity and sitting time. However, GPAQ has been shown to be reliable for use in African populations [25], and our findings are comparable with physical activity data from other African countries [32], and adds to a small body of evidence from low-and-middle-income countries. Secondly, the self-reported dietary questionnaire only considered high fat foods which have previously been associated with obesity in South Africa [24]. We observed that consumption of these foods were common amongst the participants in this study. Future studies in this population should also consider the impact of other foods which were not considered in the present investigation e.g. high carbohydrate foods and sugar sweetened drinks. Thirdly, it would have been ideal to have more regular follow-up visits of this cohort, but due to infrastructural constraints this was not possible. However, the 10-year period allowed for greater changes of the anthropometric variables than would be observed over a shorter time period thus allowing us to more easily isolate predictor variables. A further limitation was that DXA data was not available for the full cohort. However, when comparing the women for who complete DXA data was available to those for who it was not, there were no differences in any of the measured variables with the exception of age, which was slightly lower in the latter group. This suggests minimal selection bias for the group with DXA data available at both time points. Lastly, an assumption has been made that the desire for, or the acceptance of, a higher body size is the consequence of a traditional African belief in the positive aspects of obesity. Questions were not asked of the study participants to validate this assumption, although previous reports have described such beliefs [11, 14]. Comparison with a European population would have allowed us to determine whether this acceptance of a high body size was more prevalent in an African population, although other studies have shown this to be the case [9, 16].

## Conclusions

The findings of this longitudinal study demonstrate that in our cohort of black urbanised South African women time spent in vigorous physical activity was associated with smaller increases in body weight and adiposity. However, only a small percentage of subjects participated in vigorous PA. In addition, the majority of participants who were overweight or obese underestimated their body size and this was related to a smaller gain in body adiposity and a more prevalent desire to lose weight than in subjects who accurately identified their body size. Additionally, in agreement with previous studies that have reported that black South African women are more accepting of being obese [11, 14, 28], our study highlighted the existence of a further group of overweight/obese women who were content with their body size. Thus, the presence of two distinct groups of overweight/obese women within our study population, one wishing to be thinner and the other content with their body size, may reflect a clash of traditions with the former group appearing to be more closely aligned with Western values focussing on leanness and the latter group more aligned with the African ideal of overweight/obesity as the preferred body size. It is recommended that more in-depth studies of these groups are necessary to determine whether these assumptions are correct and to investigate whether obesity intervention programs would require different approaches in each of these populations.

## Supporting Information

S1 Table Comparison between body composition, lifestyle and psychosocial characteristics of black African women with and without DXA data at both time points. (DOCX)

S2 Table Comparison between baseline FID groups at baseline for follow-up body composition measures. (DOCX)

S3 Table Comparison between PAD groups at baseline for follow-up body composition measures. (DOCX)
