# Supplementary material for: The Role of Lifestyle and Psycho-Social Factors in Predicting Changes in Body Composition in Black South African Women
Source: PLoS One. 2015 Jul 14;10(7):e0132914. doi: 10.1371/journal.pone.0132914 (PMC4501844; doi:10.1371/journal.pone.0132914)
Supplement: S1 Table — (DOCX) [file pone.0132914.s001.docx]

**S1 Table. Comparison between body composition, lifestyle and psychosocial characteristics of black African women with and without DXA data at both time points**

| **Variable** | **Women with DXA at both time points (n=264)** | **Women without DXA at both time points (n=164)** |
| --- | --- | --- |
| **Age (years)** | 42.0 ± 5.50 | 39.4 ± 4.62* |
| **Body mass index (kg.m^-2^)** | 30.5 ± 6.32 | 31.3 ± 7.33 |
| **Waist circumference (cm)** | 88.3 ± 12.9 | 86.2 ± 13.6 |
| **Hip circumference (cm)** | 113 ± 12.9 | 115 ± 15.0 |
| **Sitting time (mins.wk^-1^)** | 1260 (840-1680) | 1260 (840-2100) |
| **Physical activity (mins.wk^-1^)** | 300 (140-840) | 480 (180-1440) |
| **Current smokers** | 9 (3.53%) | 4 (2.86%) |
| **Alcohol use** | 47 (18.4%) | 25 (17.6%) |
| **Feel minus ideal index (FID)** | 0.92 ± 1.53 | 1.24 ± 1.62 |
| **Perceived minus actual weight status discrepancy score (PAD)** | -0.95 ± 0.75 | -0.84 ± 0.68 |

*p<0.005; data given as mean ±SD, median (interquartile range) or n (%)
