# Supplementary material for: The Role of Lifestyle and Psycho-Social Factors in Predicting Changes in Body Composition in Black South African Women
Source: PLoS One. 2015 Jul 14;10(7):e0132914. doi: 10.1371/journal.pone.0132914 (PMC4501844; doi:10.1371/journal.pone.0132914)
Supplement: S2 Table — (DOCX) [file pone.0132914.s002.docx]

**S2 Table. Comparison between baseline FID groups at baseline for follow-up body composition measures**

| **Variable** | **Subjects who wanted to be fatter** | **Subjects who were content with their body shape** | **Subjects who wanted to be leaner** |
| --- | --- | --- | --- |
| **BMI (kg.m^-2^)** | 26.5 ± 4.90 (44) | 31.0 ± 5.65(137)^†^ | 35.5 ± 7.50 (247)^††† ***^ |
| **Waist circumference (cm)** | 87.7 ± 12.3 (43) | 94.7 ± 11.5 (134) | 103 ± 15.1 (238)^††† ***^ |
| **Hip circumference (cm)** | 105 ± 10.9 (43) | 114 ± 11.5 (133) | 123 ± 15.6 (237)^††† ***^ |
| **Fat mass (kg)** | 24.2 ± 9.0 (32) | 31.4 ± 9.53 (84) | 35.4 ± 10.1 (148)^††† *^ |
| **Fat free soft tissue mass (kg)** | 41.6 ± 7.08 (32) | 43.5 ± 6.24 (84) | 46.7 ± 7.48 (264)^††† ***^ |
| **Central adiposity (kg)** | 10.1 ± 4.34 (30) | 13.5 ± 4.69 (84) | 15.8 ± 5.32 (147)^††† *^ |
| **Peripheral adiposity (kg)** | 13.2 ± 4.75 (30) | 17.1 ± 5.51 (84) | 19.1 ± 5.71 (147)^††† *^ |

Data presented as mean ± SD (n); ^†^P<0.05, ^†††^P<0.0005 versus subjects who wanted to be fatter; ^*^P<0.05, ^***^P<0.0005 versus subjects who were content with body shape; abbreviation, body mass index (BMI)
