# Supplementary material for: The Role of Lifestyle and Psycho-Social Factors in Predicting Changes in Body Composition in Black South African Women
Source: PLoS One. 2015 Jul 14;10(7):e0132914. doi: 10.1371/journal.pone.0132914 (PMC4501844; doi:10.1371/journal.pone.0132914)
Supplement: S3 Table — (DOCX) [file pone.0132914.s003.docx]

**S3 Table. Comparison between PAD groups at baseline for follow-up body composition measures**

| **Variable** | **Subjects who underestimated actual body weight** | **Subjects who accurately perceived actual body weight** | **Subjects who overestimated actual body weight** |
| --- | --- | --- | --- |
| **BMI (kg.m^-2^)** | 34.7 ± 6.57 (316) | 29.0 ± 7.62 (103) | 23.3 ± 4.37 (9)^††† ***^ |
| **Waist circumference (cm)** | 102 ± 13.5 (307) | 90.8 ± 14.7 (97) | 82.7 ± 11.1 (9)^††† *^ |
| **Hip circumference (cm)** | 121 ± 13.9 (306) | 111 ± 15.5 (96) | 99.1 ± 11.2 (9)^††† *^ |
| **Fat mass (kg)** | 35.2 ± 9.65 (198) | 26.3 ± 9.82 (59) | 18.3 ± 9.06 (6)^††† ***^ |
| **Fat free soft tissue mass (kg)** | 46.8 ± 6.69 (198) | 40.6 ± 6.20 (59) | 32.5 ± 3.85 (6)^††† ***^ |
| **Central adiposity (kg)** | 15.8 ± 4.71 (196) | 10.6 ± 4.83 (58) | 7.28 ± 4.23 (6)^††† ***^ |
| **Peripheral adiposity (kg)** | 18.9 ± 5.49 (196) | 14.9 ± 5.41 (58) | 10.2 ± 5.16 (6) |

Data presented as mean ± SD (n); ^†^P<0.05, ^†††^P<0.0005 versus subjects who underestimated actual body weight; ^*^P<0.05, ^***^P<0.0005 versus subjects who accurately perceived actual body weight; abbreviation, body mass index (BMI)
